# Development of Alveolar Hemorrhage After Pfizer-BioNTech COVID-19 mRNA Vaccination in a Patient With Renal-Limited Anti-neutrophil Cytoplasmic Antibody-Associated Vasculitis: A Case Report

**DOI:** 10.3389/fmed.2022.874831

**Published:** 2022-04-08

**Authors:** Ken Nishioka, Shintaro Yamaguchi, Itaru Yasuda, Norifumi Yoshimoto, Daiki Kojima, Kenji Kaneko, Mitsuhiro Aso, Tomoki Nagasaka, Eriko Yoshida, Kiyotaka Uchiyama, Takaya Tajima, Jun Yoshino, Tadashi Yoshida, Takeshi Kanda, Hiroshi Itoh

**Affiliations:** ^1^Division of Endocrinology, Metabolism and Nephrology, Department of Internal Medicine, Tokyo, Japan; ^2^Apheresis and Dialysis Center, Keio University School of Medicine, Tokyo, Japan

**Keywords:** renal-limited ANCA-associated vasculitis, relapse, alveolar hemorrhage, COVID-19 mRNA vaccination, rituximab

## Abstract

Since the coronavirus disease 2019 (COVID-19) pandemic continues and a new variant of the virus has emerged, the COVID-19 vaccination campaign has progressed. Rare but severe adverse outcomes of COVID-19 vaccination such as anaphylaxis and myocarditis have begun to be noticed. Of note, several cases of new-onset antineutrophil cytoplasmic antibody-associated vasculitis (AAV) after COVID-19 mRNA vaccination have been reported. However, relapse of AAV in remission has not been recognized enough as an adverse outcome of COVID-19 vaccination. We report, to our knowledge, a first case of renal-limited AAV in remission using every 6-month rituximab administration that relapsed with pulmonary hemorrhage, but not glomerulonephritis, following the first dose of the Pfizer-BioNTech COVID-19 vaccine. The patient received the COVID-19 vaccine more than 6 months after the last dose of rituximab according to the recommendations. However, his CD19^+^ B cell counts were found to be increased after admission, indicating that our case might have been prone to relapse after COVID-19 vaccination. Although our case cannot establish causality between AAV relapse and COVID-19 mRNA vaccination, a high level of clinical vigilance for relapse of AAV especially in patients undergoing rituximab maintenance therapy following COVID-19 vaccination should be maintained. Furthermore, elapsed time between rituximab administration and COVID-19 mRNA vaccination should be carefully adjusted based on AAV disease-activity.

## Introduction

As of February 2022, 390 million cases of COVID-19, with 5.7 million COVID-19 related deaths, have been reported worldwide (1). Large clinical trials have shown that COVID-19 mRNA vaccines were sufficiently safe and effective in preventing COVID-19 (2). Besides the common adverse events following COVID-19 mRNA vaccination, including injection site reactions, fever, fatigue, headaches, and muscle pain (2), rare but severe adverse reactions such as anaphylaxis and myocarditis have been reported (3–5).

Antineutrophil cytoplasmic antibody (ANCA)-associated vasculitis (AAV) is a group of multisystem autoimmune diseases. It is characterized by production of ANCAs, directed either at proteinase 3 (PR3) or myeloperoxidase (MPO), and small to medium vessel inflammation (6). Rituximab is used as maintenance therapy for AAV and typically administered every 6 months (7). However, it is recommended that patients should wait for COVID-19 vaccination until six months elapse after the last rituximab administration (8). Several cases describing the temporal association between COVID-19 vaccination, and new-onset or relapse of AAV have already been reported (9–14). Herein, we report the first case of renal-limited vasculitis in remission using every 6-month rituximab administration that relapsed with pulmonary hemorrhage, but not glomerulonephritis, following the first dose of Pfizer-BioNTech COVID-19 vaccination.

## Case Description

**FIGURE 1:**
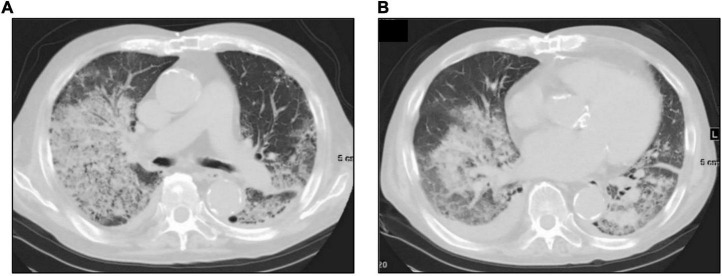
High-resolution computed tomography of the chest on admission. Chest computed tomography on admission shows diffuse “ground-glass” opacities **(A)**, consolidation, cardiac enlargement, and bilateral pleural effusion **(B)**.

**FIGURE 2:**
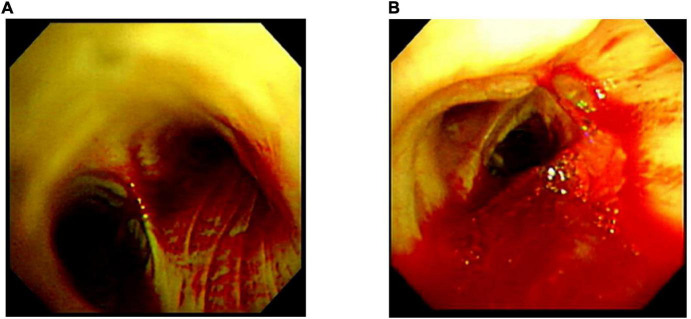
Bronchoscopic images taken prior to the initiation of plasma exchange. Bronchoscopic images taken on the 2nd admission day revealed bleeding from multiple bronchi **(A)** to vocal cord lesions **(B)**.

**FIGURE 3:**
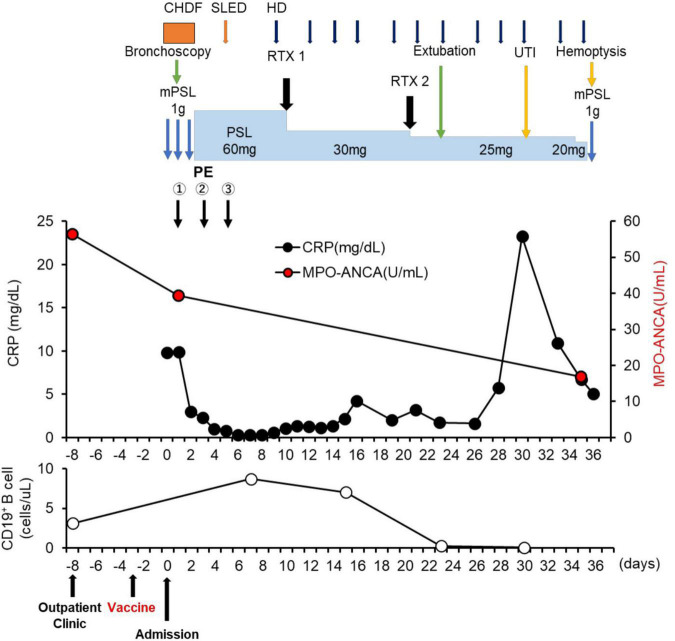
Clinical time course of the patient. CHDF, Continuous hemodiafiltration; SLED, sustained low efficiency dialysis; RTX, rituximab; mPSL, methylprednisolone; PSL, prednisolone; HD, hemodialysis; UTI, urinary tract infection.

A 74-year-old Japanese man experienced renal-limited vasculitis in 2016, and achieved remission with prednisolone and intravenous cyclophosphamide. Remission was maintained in the patient with prednisolone (10 mg/day) and azathioprine (2 mg/kg/day) until 2020 (15). Although the patient has been in clinical remission, his anti-MPO antibody remained positive. Therefore, we switched his maintenance regimen from azathioprine-based to rituximab-based therapy (375 mg/m^2^ every 6 months) (7, 16). The patient received his third rituximab dose in mid-April 2021. Eight days before admission in late October, his serum creatinine level was stable at 3.15 mg/dl. Given that urinalysis showed no red blood cells and stable proteinuria at 0.61 g/gCr, his renal-limited vasculitis was confirmed to be in remission. Three days before admission, he received his first dose of the Pfizer-BioNTech COVID-19 vaccine. The patient had no allergies to the drugs or documented history of COVID-19. On the day of the first vaccination, the patient developed cough with productive sputum, and dyspnea. Since dyspnea worsened and hemoptysis appeared over 3 days, he presented to our emergency department. His oxygen saturation was 68% on room air, blood pressure was 90/41 mmHg, and heart rate was 90 beats per min. Reverse transcription-polymerase chain reaction testing for severe acute respiratory syndrome coronavirus 2 (SARS-CoV-2) RNA from nasopharyngeal swabs yielded negative results. Laboratory test results on admission showed acute kidney injury in chronic kidney disease with serum creatinine levels of 6.43 mg/dl, high inflammatory reaction with C-reactive protein of 9.84 mg/dl, and brain natriuretic peptide of 4031.8 pg/ml. Urinalysis showed no urinary blood cells and random urinary protein-creatinine ratio was 0.57 g/gCr, which was comparable with the result at his last outpatient visit. Serum complement levels and immunological workup including rheumatoid factor, anti-glomerular basement (GBM) and anti-double-stranded DNA antibodies were unremarkable (Table 1). High-resolution computed tomography of the chest showed diffuse “ground-glass” opacities and consolidation in both lungs and cardiac enlargement with bilateral pleural effusion. These findings suggested that the patient had alveolar hemorrhage complicated by cardiogenic pulmonary edema (Figures 1A,B). Thus, he was suspected to have vasculitis relapse with diffuse alveolar hemorrhage, but not glomerulonephritis. Particularly, no tumor was detected on computed tomography. Blood and sputum culture results were negative. The patient received mechanical ventilation with positive end-expiratory pressure, and treatment was initiated empirically with intravenous methylprednisolone pulse for 3 days, followed by prednisolone 1 mg/kg daily. His urinary output was minimal, requiring continuous venovenous hemodiafiltration with intravenous catecholamine support. Bronchoscopy with bronchoalveolar lavage revealed hemosiderin-laden macrophages in hemorrhage fluid, leading to a diagnosis of diffuse alveolar hemorrhage (Figures 2A,B). Therefore, plasma exchange was performed thrice every other day, and rituximab was administered weekly at 375 mg/m^2^ in two doses for remission reinduction of AAV on the basis of clinical presentation of alveolar hemorrhage. His hemoptysis and alveolar hemorrhage on bronchoscopy improved significantly, and he withdrew from mechanical ventilation. Renal biopsy was not performed due to the patient critical state. However, considering that neither urinary red blood cells nor increase in urinary protein was observed over the treatment period, acute deterioration in chronic kidney disease requiring hemodialysis was not likely attributed to AAV relapse but to cardio-renal syndrome (type 5). The patient remained on hemodialysis, whereas his general condition remarkably improved. However, the patient developed urinary tract infection, and rituximab was suspended for infectious control. He experienced recurrent hemoptysis and died of progressive respiratory failure within a day (Figure 3).

**TABLE 1:** Clinical laboratory data of the patient.

	Days relative to first vaccine dose		
	−5	+3	Reference range
Serum sodium, mEq/l	138.3	139.7	138-145
Serum potassium, mEq/l	5.3	5.8	3.6–4.8
Serum chloride, mEq/l	105	105	101–108
Serum bicarbonate, mEq/l	–	19.9	22–26
SUN, mg/dl	59.5	92.1	8–20
Serum creatinine, mg/dl	3.15	6.43	0.65–1.07
C-reactive protein, mg/dl	1.56	9.84	<0.14
Brain natriuretic peptide, pg/ml	1798.6	4031.8	<18.4
Hemoglobin, g/dl	10.7	9.8	13.7–16.8
MPO-ANCA, U/ml	56.4	39.3	0–3.4
PR3-ANCA, U/ml	–	<1.0	0–3.4
anti-GBM antibody, U/ml	–	<2.0	0–2.9
ANA	–	<40	0–39
anti-dsDNA antibody, U/ml	–	4.0	0–12
RF, U/ml	–	9	0–15
C3, mg/dl	–	79	73–138
C4, mg/dl	–	23	11–31
**Urinalysis**			
Specific gravity	1.015	1.008	1.005–1.030
Blood	Negative	Negative	Negative
Glucose	Negative	Negative	Negative
Ketones	Negative	Negative	Negative
Leukocyte esterase	Negative	Negative	Negative
Nitrite level	Negative	Negative	Negative
Protein, g/gCr	0.61	0.57	<0.15
RBCs per HPF	Negative	Negative	0–4
WBCs per HPF	1–4	Negative	0–4

*GBM, glomerular basement membrane; ANA, antinuclear antibody; dsDNA, double-stranded deoxyribonucleic acid; RF, rheumatoid factor; RBCs, red blood cells; HPF, high-power field; WBCs, white blood cells; –, unmeasured.*

## Discussion

Herein, we describe the case of AAV relapse with alveolar hemorrhage after the first dose of COVID-19 mRNA vaccination. To date, several cases of *de novo* ANCA vasculitis and one case of AAV relapse after remission following the second dose of COVID-19 mRNA vaccination have been reported (9–14). Our case was unique in that alveolar hemorrhage, but not glomerulonephritis, developed as a result of renal-limited vasculitis relapse shortly after receiving the first dose of Pfizer-BioNTech mRNA COVID-19 vaccine. This case also represents a dilemma of waiting for COVID-19 mRNA vaccination until 6 months have elapsed after the last dose of rituximab, which might exacerbate AAV activity and increase the risk for AAV relapse.

AAV is an autoimmune disease that could lead to worse COVID-19 outcomes, especially when patients are on 10 mg/day or more of prednisolone, cyclophosphamide, and rituximab (17, 18). Since vaccines appear to prevent SARS-CoV-2 infection and further reduce the risk of disease aggravation and death, the American College of Rheumatology recommends COVID-19 mRNA vaccines for patients with rheumatic and musculoskeletal diseases (19). Our patient was treated with prednisolone 10 mg/day and rituximab for renal-limited vasculitis as maintenance therapy. Therefore, COVID-19 vaccination was strongly recommended for him.

The mechanism for AAV recurrence after vaccination remains enigmatic. However, some reports have described cases of AAV developed after influenza vaccination (20–22), which raised the possibility that an immune response to either the vaccine antigen or one of the excipients in the vaccine might have caused AAV (22). Of note, peripheral blood mononuclear cells extracted from patients in remission of PR3-ANCA positive vasculitis significantly produced more PR3-ANCA in response to influenza vaccines containing RNA in an *ex vivo* study compared to healthy controls (23).

Billions of people worldwide have received COVID-19 vaccinations (1). Rare but severe adverse events following these vaccinations, such as new-onset or recurrent glomerulonephritis are emerging (24). Similar to influenza vaccines, COVID-19 vaccination may induce dysregulation of immune response to the spike protein or mRNA of SARS-CoV-2 (9–14), giving rise to AAV, as the previously postulated mechanism for SARS-CoV-2 infection (25). It is suggested that antibodies against SARS-CoV-2 spike glycoprotein could cross-react with autoimmune target proteins (26), triggering pre-existing or underlying immune dysregulation in a specific host, ultimately jeopardizing disease flares such as AAV (27). One possibility is that our patient might have had a dormant underlying autoimmune disease in the lungs, which became apparent due to an aberrant immune response provoked by the COVID-19 vaccine. To our best knowledge, there has been only one case of AAV in remission involving the kidney and lung that recurred after COVID-19 vaccination (14). In our case, it is difficult to prove a causal relationship between vaccination and AAV relapse with severe alveolar hemorrhage. Indeed, our patient had no elevated titer of anti-MPO antibody on admission compared with that result of his last outpatient visit (titer on admission: 39.3 U/ml; titer at the last visit: 56.4 U/ml; normal: <3.4 U/ml). Although MPO-ANCAs are known to be pathogenic (28), disease activity of AAV is not always correlated with MPO-ANCA titers, but rather with MPO-ANCA affinity (29, 30). However, after initiating treatment of AAV relapse, the titer of anti-MPO antibody decreased coinciding with improvements in pulmonary hemorrhage (Figure 3), which is similar to a previous case report (31). In addition, Sharma et al. reported a unique case demonstrating that diffuse alveolar hemorrhage occurred 8 h after the first dose of COVID-19 mRNA vaccination without any evidence of immunological complications such as elevation of anti-MPO antibody titers (32). Infection and tumor, two major inciting factors for AAV relapse (33–35) and other immunological underlying diseases for pulmonary-renal syndrome, such as anti-glomerular basement membrane (GBM) disease: Goodpasture syndrome, systemic lupus erythematosus, and dual ANCA and anti-GBM positive disease (36) have been ruled out. Therefore, we suspect that these adverse events in our patient were not mere coincidences. In particular, the time course for onset of vaccine-related symptoms was consistent with these in previous reports that varied from a few hours after the first dose to 6 weeks after the second dose (24). In addition, we cannot exclude the possibility that AAV recurrence might have been related to the elapsed time since the last dose of rituximab (6 months). Importantly, it is recommended that 6 months should be elapsed between rituximab administration and COVID-19 vaccination (8). In our case, CD19^+^ B cell counts increased from 3.1 cells/μl 5 days before vaccination to 8.7 cells/μl on day 7 after admission (Figure 3). Therefore, AAV in remission with rituximab, as in this case, may be susceptible to relapse after COVID-19 vaccination. Pertinently, the recommendation highlights the possibility that vaccination early after rituximab administration might sufficiently induce protective effects against COVID-19 infection by cellular immunity (8). Therefore, earlier administration of vaccine after rituximab should be carefully considered to prevent CD19^+^ B cell repletion and AAV relapse.

A limitation to this case report is that we did not demonstrate causality between COVID-19 mRNA vaccination and AAV relapse. This study also did not determine the mechanisms for renal-limited vasculitis recurrence that occurred with alveolar hemorrhage but not glomerulonephritis.

Clinicians should be aware of a possible causal relationship between AAV relapse and COVID-19 mRNA vaccination and might have to carefully adjust elapsed time between rituximab administration and COVID-19 vaccination based on AAV disease-activity.

## Data Availability Statement

The original contributions presented in the study are included in the article/supplementary material, further inquiries can be directed to the corresponding author.

## Ethics Statement

Written informed consent was obtained from the individual(s) for the publication of any potentially identifiable images or data included in this article.

## Author Contributions

KN and SY wrote the manuscript. KN, SY, IY, NY, DK, KK, MA, TN, EY, KU, TT, JY, TY, TK, and HI took clinical care of the patient. All authors have read and approved the final manuscript.

## Conflict of Interest

The authors declare that the research was conducted in the absence of any commercial or financial relationships that could be construed as a potential conflict of interest.

## Publisher’s Note

All claims expressed in this article are solely those of the authors and do not necessarily represent those of their affiliated organizations, or those of the publisher, the editors and the reviewers. Any product that may be evaluated in this article, or claim that may be made by its manufacturer, is not guaranteed or endorsed by the publisher.
